# Effect of Dietary Supplementation on Body Composition, Pulmonary Function and Health-Related Quality of Life in Patients with Stable COPD

**Published:** 2016

**Authors:** Naushad Ahmad Khan, Naresh Kumar, Mradul K Daga

**Affiliations:** Department of Medicine, Maulana Azad Medical College, New Delhi, India

**Keywords:** COPD, Quality of life, Exercise capacity, Pulmonary function, Dietary supplementation

## Abstract

**Background:**

Malnutrition is very common in patients with chronic obstructive pulmonary disease (COPD). Nutritional supplementation improves the patient’s nutritional status by increasing the energy intake and providing anti-inflammatory elements which can relieve the patient’s symptoms and delay the disease progression. This study sought to determine if energy and protein supplementation improves physical function, pulmonary function and health-related quality of life (HRQL) in stable COPD patients.

**Materials and Methods:**

The study was carried out in an outpatient setting on 60 stable COPD patients over a period of one year. Patients were randomized to intervention group (n=30), receiving supplemental nutrition in the form of additional protein and carbohydrates or control group (n=30), receiving only the usual standard diet. Lung function, body mass index (BMI), exercise capacity (6-minute walk test or 6MWT), mid-upper arm circumference (MUAC) and skin fold thickness (SFT) were evaluated, and clinical assessment was carried out at baseline and after completion of 12 weeks. The HRQL was assessed using Seattle obstructive lung disease questionnaire.

**Results:**

Twelve weeks of dietary supplementation resulted in a significant increase in weight and BMI of patients in the intervention group in comparison to the control group (P<0.005). Significant improvement was also observed in 6MWT and HRQL scores after nutritional intervention (P=0.002 and P=0.001, respectively). However, difference in MUAC, SFT and serum protein level after 12 weeks of follow up was not significant in any of the two groups. There was a similar degree of lung function improvement in both groups although it was not statistically significant.

**Conclusion:**

Nutritional supplementation with high protein and energy diet during 12 weeks of intervention improved body weight and composition, exercise capacity and quality of life in stable COPD patients.

## INTRODUCTION

Chronic obstructive pulmonary disease is an important health problem across the globe. The disease is characterized by persistent airflow obstruction which is progressive resulting from inflammation and remodeling of the airways and lungs stimulated by exposure to toxins mainly due to a history of cigarette smoking (1). It has been recognized as the third leading cause of death and continues to be the major cause of mortality and morbidity worldwide (2).

According to the World Health Organization consensus, 4.8% of the world population was diagnosed with COPD in 2010 (3). In 2005, more than 3 million people died of COPD accounting for 5% of all deaths globally and it is expected to become the third leading cause of death by 2030 (4). The current prevalence of COPD is not uniformly available in the Indian population. Although studies done by Murthy and Sastri (5) and Jindal (6). have reported the average prevalence rate of 5% in the adult population, higher rates have been reported in smokers, males, and rural areas.

Malnutrition is very common in patients with COPD. Insufficient energy intake and systematic inflammation lead to malnutrition in these patients (7). Common clinical systemic manifestations are changes in body composition, metabolism and immune status which often lead to weight loss, dyspnea, fatigue, reduced exercise tolerance, poorer prognosis, increased susceptibility to infections and impaired quality of life independent of pulmonary function (8).

Weight loss in COPD may be due to an increased energy expenditure imbalance caused by inadequate dietary intake which leads to reduction of diaphragmatic muscle mass and depressed diaphragm contractility (9). Resting energy expenditure (REE), total daily energy needed, systemic inflammation and tissue hypoxia have been reported to be elevated in stable COPD patients and are considered to be a part of pathophysiologic mechanisms for weight loss (10, 11). Patients with COPD experience gradual and progressive loss of skeletal muscle mass which not only affects their respiratory function but also causes muscle fatigue. This may further lead to reduction in exercise capacity and the ability to work (12). Malnourished patients demonstrate more gas trapping, lower diffusing capacity and a reduced exercise performance than non-malnourished patients with similar pulmonary mechanics (13).

Several attempts have been made to find an association between malnutrition and impaired pulmonary function in patients with COPD (14–16). However, the exact causal relationship between malnutrition and COPD is difficult to establish and not clear yet. Malnutrition in COPD may be the consequence of worsened disease severity leading to compromised nutritional intake and reduced physical activity (muscle atrophy) and may alternatively be responsible for the wasting of muscles involved in breathing due to the progressive nature of COPD (17). Malnourished COPD patients also have been reported to score worse than non-malnourished patients on a respiratory disease specific quality of life questionnaire (17,18).

The effect of nutritional support in malnourished patients has also been controversial. Several attempts have been made to reverse the weight loss and muscle wasting and improve respiratory and limb muscle function by administration of oral nutritional therapy (19–21) in stable COPD patients. However, several studies have also reported that nutritional intervention alone has little or no effect on improving pulmonary function parameters, functional exercise capacity and anthropometric characteristics (22–24).

These contradictory results show that confusion remains about whether there is a need to identify and treat malnutrition in COPD patients. These contrasting conclusions may have dampened interest in the field; however, it is hoped that some positive findings of the previous studies will encourage the need to undertake further work, including an examination of the interactions that might exist between nutritional supplementation and factors such as malnutrition, inflammatory status, graded physical activity and HRQL in stable COPD patients. The literature has shown equivocal benefits from long-term (>4weeks) nutrition supplementation program in COPD. There is a paucity of Indian specific data regarding the assessment of nutritional intervention or role of dietary supplementation in stable COPD patients. To the best of our knowledge, none of the Indian studies so far have assessed the effects of dietary supplementation on patient – center outcome including quality of life. Hence, the present study was undertaken to assess the specific impact of nutritional supplementation and its effect on anthropometrics, spirometry (pulmonary function tests), exercise capacity and HRQL in stable COPD outpatients.

## MATERIALS AND METHODS

### Study Population

This was a prospective interventional study with 60 clinically stable patients of COPD attending chest clinic of our hospital. The study was done at Maulana Azad Medical College and associated Lok Nayak Hospital, New Delhi over a period of one year. The study had the following inclusion criteria:

1. A Clinical diagnosis of COPD confirmed according to the Global Initiative for Chronic Obstructive Lung Disease guidelines (1).
2. Clinically stable COPD patients who did not have acute exacerbation in the past three months and were ≥35 years.

The diagnosis of COPD was confirmed as a post-bronchodilator forced expiratory volume in 1 second (FEV1)/forced vital capacity (FVC) ratio of <0.7. The severity of the disease was graded according to the Global Initiative for Chronic Obstructive Lung Disease guideline. Severe COPD was defined as predicted FEV1: 30% ≤ FEV1 < 50%; and very severe: FEV1 < 30% or FEV1 < 50% and chronic respiratory failure (1). All patients were ex-smokers and receiving optimal medical treatment including inhaled long acting anticholinergics, inhaled corticosteroids/β2-agonists and theophylline. None of the patients received systemic steroids or were dropped out due to acute exacerbation.

Patients suffering from any other chronic illness such as diabetes mellitus, chronic liver disease, tuberculosis, lung cancer or malignancy were excluded. The study had approval from institutional ethics committee and all subjects gave their written consent to participate in the study. All the patients were evaluated and a complete history was taken including age, sex, smoking history and physical examination. Smoking rate (Packs/year) was calculated from the average number of cigarettes smoked in one day over one year. Pulmonary function test, nutritional assessment including anthropometric measurement and serum protein levels, 6MWT and HRQL assessment by the Seattle obstructive Lung disease questionnaire were done at the start of the study and all these measurements were repeated after 12 weeks of nutritional intervention or usual care.

### Assessments

**Pulmonary function tests**: Spirometry was done on the first day of admission or in the outpatient clinic for stable COPD patients. The FEV1 and FVC were measured with a calibrated spirometer (Spirolab III, MIR, Italy; portable pulmonary function apparatus). The FEV1, FVC and FEV1/FVC ratio were calculated. All the measurements were done in a sitting position by the same technician to ensure consistency of the technique. The best test from three consecutive tests was accepted. FEV1, FVC, FEV1/FVC were measured according to American Thoracic Society (ATS) criteria (25).

**Anthropometrics, body composition, exercise capacity and dyspnea assessments:** At the clinic visit, information about demographics and a detailed medical history were obtained. Height was measured in centimeters and weight was measured by a calibrated scale in kilograms with subjects not wearing shoes. The BMI was calculated as weight (in kilograms)/height^2^ (in meters). MUAC of the right arm was taken using a flexible measuring tape and SFT was measured in triplicate using the Holtain skin fold caliper over the triceps muscle of the right arm of the patients. Functional exercise capacity was measured with 6MWT in accordance with the ATS recommendations (26). The test was performed in a level, covered hospital corridor of approximately 50 m in length. Three tests were performed and the test with the maximum six-minute walking distance (6MWD) was considered for analysis. Each patient received standard instructions and encouragement during the test. The magnitude of dyspnea was assessed using the modified scale of Medical Research Council (mMRC) (27). Patients were asked about their perceived breathlessness and were then classified into the MMRC five dyspnea grades (0: minimum to 4: maximum).

**Health related quality of life:** HRQL was assessed using the brief self-administered instrument named Seattle obstructive lung disease questionnaire a 29-question instrument developed to measure physical function, emotional function, coping skills, and treatment satisfaction (21). Seattle obstructive lung disease questionnaire translated to Hindi language was earlier validated in our department to study the impact of anabolic steroids in patients with chronic obstructive lung disease in 30 patients. The Hindi version of the questionnaire was developed following the forward-backward translation procedure with independent translations and counter-translations.

**Biochemical Indices:** Serum protein concentrations and hemoglobin were measured at baseline and after eight weeks of supplementation in all patients. A stable regime of inhaled and oral bronchodilators and inhaled steroids was maintained during the study. The use of systemic steroids was not allowed during the study period.

### Study design

A single-blind randomized, parallel group, design was used. After baseline assessment, the eligible patients were allotted by computer generated random numbers to one of the two groups and followed up for 12 weeks. A total of 65 patients were screened, out of which 60 patients were recruited and randomized into two groups of intervention and control (n=30 in each group). Those who were dropped out or in whom the investigations could not be completed were excluded leaving 3 and 2 patients, in intervention group and control group respectively.

**Intervention group:** In addition to their usual diet, patients in the intervention group ((n=30) were given supplemental nutrition in the form of commercial protein powder PROTENIX, 30 g per day (Manufactured by Wockhardt pharmaceuticals Pvt. Ltd). This provides 90 Kcal of energy in 2 tps servings and comprises of 12 g (55%) carbohydrates, 10 g (45%) protein and 0% fat. for a period of 12 weeks along with standard treatment for COPD. In the follow up period, it was assured in this group that the supplement was taken in addition to their usual meal and it did not decrease their total intake.

**Control group:** Patients in the control group (n=30) were left on their usual diet. These patients received standard treatment for COPD which included inhaled ipratropium plus salmeterol/ formoterol with inhaled fluticasone/budesonide.

Inhaled salbutamol was allowed on an as-needed basis. Exacerbations, if any, were planned to be managed by additional bronchodilators, oral steroids and antibiotics if necessary. All the anthropometric measurements, serum protein level assessment, 6MWT and HRQL assessment were repeated after 12 weeks in both groups. Adverse events were recorded.

### Statistical Analysis

Data from the treatment and the control groups were analyzed with the statistical software package, SPSS 17.0 (Statistical Package for Social Sciences, SPSS Inc., USA). A normality test was performed to assess the normality of distribution of the data. Descriptive statistics were carried out for all parameters. Independent student’s *t*-test was used to look for differences in age, packs/year smoked, anthropometric measurements, pulmonary function parameters, exercise capacity, and HRQL scores for treatment and control groups for comparison of baseline characteristics. Paired t-test was used to compare anthropometric measurements, exercise capacity and HRQL scores before and after the treatment intervention. Furthermore, measurement values were expressed as mean ± standard deviation except when means ± SEMs are used in figures and a value of P<0.05 was considered statistically significant. The Pearson’s correlation test was used to assess the relationships between variables.

## RESULTS

### Baseline evaluation:

Totally 60 patients were enrolled in the study and were evaluated prospectively with 30 patients in each group. The clinical and demographic characteristics were comparable (age, height, weight and smoking) in both groups (Table 1). The mean age of the intervention group was 55.03±10.41 yrs. and that of control group was 53.33±10.76 yrs. Fifty-eight patients out of 60 (96%) were smokers in this study. No subject attended a pulmonary rehabilitation program while participating in the study. Baseline characteristics of the patients are summarized in Tables 1 and 2. The mean arterial blood gases was normal. No baseline differences between the intervention and the control group were seen with regards to BMI, arterial oxygen tension, FEV1, 6MWD, HRQL, Serum protein, MUAC, and SFT at baseline (Table 2).

**Table 1.** Baseline demographics and clinical characteristics of the subjects entering the study (n=60)

**Preliminary characteristics**	**Intervention group**	**Control group**
**No. of Patients**	30	30
Age in years (mean±SD)	55.03±10.41	53.33±10.76
Male/ female ratio	26/4	28/2
Height (cm)	163.53±10.54	161.70±6.93
Weight (kg)	51.93±10.24	52.13±11.59
BMI	17.81±3.32	18.13±4.83
**Smoking History**		
Smokers	30	28
Nonsmokers	0	2
Packs/year (mean±SD)	21.76±4.68	20.80±4.88
**Severity of COPD**		
Mild (I)	4	3
Moderate (II)	10	11
Severe (III)	14	13
Very Severe (IV)	2	3
FFMI (Kg/m^2^)	15.4±1.4	15.9±1.6
Arterial PO_2_	63.8±4.24	62.7±4.46
Arterial PCO_2_	43.4±3.4	40.3±2.3^*^

Data are presented as group mean ± (SD) values

* Statistically significant compared to intervention group (P value <0.05; independent Student’s *t* test) FFMI: Fat free mass index PaO_2_: Arterial oxygen pressure; PaCO_2_: Arterial carbon dioxide pressure; SaO_2_: Oxygen saturation;

**Table 2.** Difference between the studied parameters in the intervention group and control group at baseline

Parameters	Intervention Group (n=30)	Control Group (n=30)	P-value

	Baseline		
Weight (kg)	51.93±10.24	52.13±11.59	0.944
BMI (kg/m^2^)	17.81±3.32	18.13±4.83	0.727
MUAC	25.25±2.33	24.45±2.12	0.172
SFT	9.98±0.41	9.99±0.64	0.906
S.protein	6.35±0.55	6.14±0.43	0.114
FEV_1_%	52.87±9.48	51.31±13.73	0.962
FVC	73.61±11.60	71.58±13.36	0.480
FEV_1_/FVC	69.44±7.88	67.62±7.88	0.413
6MWT(mt)	301.00±19.13	297.16±16.90	0.414
HRQL	112.13±4.05	109.60±5.28	0.091

BMI: Body Mass Index; FEV1: Forced expiratory volume; FVC: forced vital capacity; MUAC: Mid upper arm circumference; SFT: Skin fold thickness; 6MWT: Six- minute walk test; HRQL: Health related quality of life

Data are presented as group mean (SD) values.

### Post intervention evaluation:

There was a significant increase in weight and BMI of the patients in the intervention group but none in control group after 12 weeks of nutritional supplementation. In this study there were 12 patients in the study group and 18 patients in the control group with BMI < 18.5 Kg/m^2^ meaning that 30 out of 60 patients were underweight i.e., incidence of malnutrition in this study was 50%.

The difference in the mean changes of studied parameters within and between the groups at the end of 8 weeks is shown in Table 3. The improvement in 6MWD and HRQL scores was found to be highly significant in the intervention group in comparison to the control group (P<0.05). The HRQL scores and 6MWD also increased after 12 weeks in the control group but failed to attain statistical significance. The differences observed in serum protein, MUAC and SFT between the groups after 12 weeks were not significant (Tables 3 and 4). Slight improvements were observed in the lung function parameters (FEV1, FVC, and FEV1/FVC) in both groups at the end of 12 weeks, although these changes were not statistically significant (Table 3). Also, independent t-test used to compare differences in lung function parameters post-supplementation vs pre-supplementation did not show a significant change between the groups at the end of the study period (Table 4).

**Table 3.** Differences in the studied parameters before and after the intervention in the intervention and control groups (within group changes)

**Parameters**	**Intervention group (before)**	**Intervention group (after)**	**P value**	**Control group (before)**	**Control group (after)**	**P value**
**Weight (kg)**	51.93±10.24	53.41±10.49	0.021	52.13±11.59	51.96±11.37	0.714
**BMI (kg/m^2^)**	17.81±3.32	18.41±3.52	0.034	18.13±4.83	18.13±3.93	0.668
**MUAC**	25.25±2.33	25.30±2.32	0.852	24.45±2.12	24.41±2.08	0.486
**SFT**	9.98±0.41	9.98±0.41	0.982	9.99±0.64	10.00±0.66	0.326
**S. Protein**	6.35±0.55	6.50±0.54	0.177	6.14±0.43	6.10±0.37	0.232
**FEV 1%**	52.87±9.48	55.84±13.04	0.095	50.31±13.73	51.90±14.17	0.147
**FVC**	73.61±11.60	76.07±14.75	0.771	71.58±13.36	73.01±13.89	0.142
**FEV1/FVC**	69.44±7.88	70.57±8.53	0.433	64.62±7.88	67.59±10.10	0.006
**6MWT (m)**	301.00±19.13	322.16±20.83	0.001	297.16±16.90	299.16±14.13	0.541
**HRQL**	112.13±4.05	134.80±6.41	0.001	109.60±5.28	112.16±4.84	0.007

BMI: Body mass index; FEV1: Forced expiratory volume; FVC: Forced vital capacity; MUAC: Mid upper arm circumference; SFT: Skin fold thickness; 6MWT: Six-minute walk test; HRQL: Health related quality of life

Data are presented as group mean (±SD) values.

**Table 4.** Comparison of the studied parameters in the intervention and control groups at the end of the study

**Parameters**	**Intervention group (n=30)**	**Control group (n=30)**	**P-value**

	**END OF THE STUDY**		
**Weight (kg)**	53.41±10.49	51.96±11.37	0.006
**BMI (kg/m^2^)**	18.41±3.52	18.13±3.93	0.980
**MUAC**	25.30±2.32	24.41±2.08	0.338
**SFT**	9.98±0.41	10.00±0.66	0.822
**S. Protein**	6.50±0.54	6.10±0.37	0.008
**FEV 1%**	55.84±13.04	49.90±14.17	0.163
**FVC**	76.07±14.75	73.01±13.89	0.639
**FEV1/FVC**	70.57±8.53	64.59±10.10	0.008
**6MWT(m)**	322.16±20.83	298.16±14.13	0.002
**HRQL**	134.80±6.41	112.16±4.84	0.001

BMI: Body mass index; FEV1: Forced expiratory volume; FVC: Forced vital capacity; MUAC: Mid-upper arm circumference; SFT: Skin fold thickness; 6MWT: Six-minute walk test; HRQL: Health related quality of life

Data are presented as group mean (SD) value

**Figure 1.**
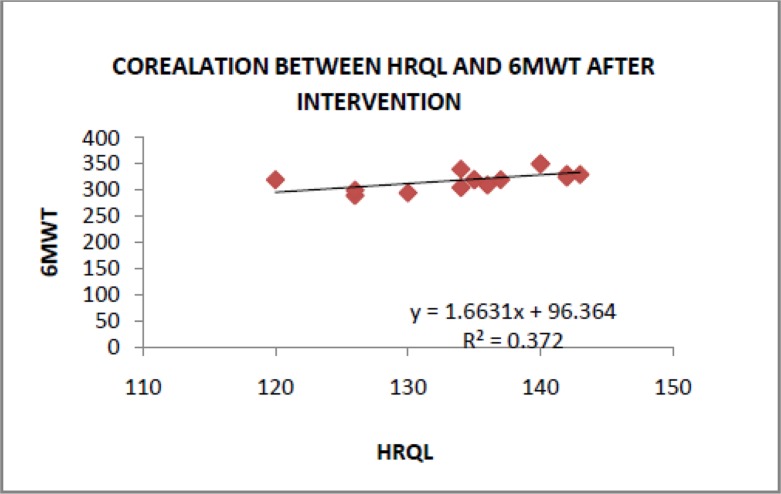
Correlation between the significant functional parameters (HRQL, 6MWT) observed in the intervention group after 12 weeks. The Pearson’s correlation test was used to calculate r values.

The treatment was well tolerated and no adverse events were recorded. None of the patients had an acute exacerbation requiring emergency room treatment or hospitalization. There was no impact on lipid profile, liver function tests, renal function or blood pressure. The intervention group tolerated the high-protein, high-calorie diet without any complication. At the end of the study period, a significant difference between the study and control groups was observed in the 6MWT and HRQL (P< 0.05, Table 4). These parameters were also found to be positively correlated in response to nutritional supplementation at the end of the study (Table 5 and Figure 1).

**Table 5.** Correlation of the functional parameters (BMI, 6MWT, HRQL, FEV1, serum protein) observed in the intervention group after 12 weeks

**Parameters**	**Correlation**	**BMI**	**6MWT**	**HRQL**	**FEV1%**	**S. Protein**
**BMI**	Pearson Correlation	1	0.331	0.483	−0.242	0.111
	Sig. (2-tailed)		0.229	0.068	0.386	0.694
**6MWT**	Pearson Correlation	0.331	1	0.610^*^	0.152	0.212
	Sig. (2-tailed)	0.229		0.016	0.589	0.449
**HRQL**	Pearson Correlation	0.483	0.610^*^	1	−106	0.251
	Sig. (2-tailed)	0.068	0.016		0.708	0.367
**FEV1%**	Pearson Correlation	−0.242	0.152	−106	1	0.005
	Sig. (2-tailed)	0.386	0.589	0.708		0.985
**S. Protein**	Pearson Correlation	0.111	0.212	0.251	0.005	1
	Sig. (2-tailed)	0.694	0.449	0.367	0.985	

* Correlation is significant at the 0.05 level (2-tailed).

## DISCUSSION

The relationship between the weight loss and COPD is well recognized in the literature. However, the clinical impact of nutritional supplementation and dietary habits on functional outcome of disease and its progression remains to be elucidated and is an area for scientific research. To the best of our knowledge, the current study is the first to look into the effect of nutritional supplementation on anthropometrics and pulmonary and functional outcomes in stable COPD patients in the Indian population. The results of our study suggest that nutritional support therapy plays an important role in management of malnourished COPD patients. This is evident by the findings of the current study which showed significant improvement in functional outcomes. Nutritional supplementation was successful in increasing weight, exercise capacity and HRQL of our patients. Our data demonstrated a significant increase in weight and BMI of the patients in the intervention group after nutritional intervention.

Previously conducted studies have shown that approximately 25% of COPD patients may be malnourished and almost 50% of them admitted to the hospital had evidence of malnutrition advocating the fact that malnutrition remains a common problem in such patients (17, 18). Studies have shown lean body mass depletion in COPD to be associated with an altered health status and increased mortality (28,29). It was also shown that muscle mass had strong impact on mortality in advanced COPD (30). A review of nutritional intervention suggested to examine whether it is possible for depleted COPD patients to gain weight and rebuild muscle mass with adequate nutritional support (24). Otte et al. (31) reported that BMI served as a simple and accurate indicator of nutritional status in a substantial number of stable COPD patients with nutritional abnormalities. They showed an improvement of 1.5 kg in the intervention group over a period of 13 weeks. Wilson et al. (15) found that patients gained weight when given sufficient calories in excess of their needs. They did a study on admitted COPD patients for 3 weeks and reported significant weight gain. Some of the studies on nutritional intervention in stable COPD patients have failed to achieve significant improvement in anthropometric outcomes. A study done by Lewis et al. (32) did not report a significant improvement in BMI following nutritional intervention. This may be due to the fact that the duration of supplementation was not long enough and dietary intake was not increased adequately in such patients.

Pulmonary function parameters did not improve significantly after nutritional intervention in our study. Otte et al, (31) and Wilson et al. (15) did not find any improvement in pulmonary function despite significant weight gain in their studies. Ganzoni et al. (33) conducted a longer period study of over 12 months but the improvement in FEV1 was not significant in their study. Efthimiou et al, (20) did not report a significant improvement in lung function either. The failure to achieve any significant improvement by previous studies including ours may be due to the fact that malnourished patients are prone to periodic exacerbations which can further cause deterioration of lung function; also, exposure to air pollution may have caused minor changes in the lung function. Moreover, the duration of our study was 12 weeks during which the patients might not have achieved a high nutritional intake sufficiently above the energy expenditure to achieve improvement in respiratory muscles.

We did not observe significant improvement in MUAC, SFT or serum protein level in any of the groups in our study. Studies by Lewis et al, (32) and Sridhar et al, (34) also showed no significant improvement in MUAC and SFT in patients. Otte et al. (31) showed significant improvement in SFT after 13 weeks of nutritional supplementation. Song et al. (35) showed a significant improvement in weight and serum protein level. The absence of response in MUAC and SFT to nutritional support in our study probably reflected the multifactorial mechanisms in COPD by which an increased energy expenditure is not balanced by an adequate dietary intake. Also, the duration of supplementation was only 12 weeks in our study; whereas, studies which showed significant improvement were done for much longer period (13 weeks or more) on admitted patients.

Several studies have examined the effect of nutritional supplementation on improving exercise capacity assessed by the 6MWT. Efthimiou and colleagues (20) reported significant improvements in the oral nutritional supplementation group but not in the control group as assessed by 6MWD. They reported a significant improvement by 53m (+12%) in the intervention group but not in the control group (+1m ; +1.4%). Similar results were reported by De Letter et al (36) (+35.4m; +11.4%). Also, Steiner et al. (37) showed that exercise capacity measured by 6MWT increased to a greater extent in the intervention than in the control group. However, the difference was not statistically significant. Improvements in the walking capacity (6MWT) mentioned in different studies may be attributed to increased carbohydrate ingestion following nutritional supplementation.

Although the carbohydrates remain the rich source of energy and provide endurance during exercise, they have a limited intramuscular storage. Otte et al. (31) did not find significant changes in the 12-minute walking distance in either supplementation or control group. In our study, we reported a significant improvement in the supplementation group compared to the control group in 6MWD after 12 weeks (+21m, P =0.001 vs +2m ; P =0.541) Our data were in agreement with the observations by the above mentioned researchers by showing significant improvement in 6MWT performance in the group receiving nutritional supplement.

HRQL scores improved significantly in the intervention group in the present study. Older studies have evaluated changes in HRQL using different scales. During the 3 months of dietary supplementation, Efthimiou et al. (20) did note improvement in breathlessness and general well-being that fell gradually over the subsequent 3 months. In contrast, Otte et al. (31) (using a different scale) did not identify changes in well-being associated with supplementation. Using the St. George Respiratory Questionnaire recently, Gurgun and collegues (38) demonstrated an improvement in symptom score and total score in the group receiving nutritional supplement. A recent study by Daga et al, (39) also showed significant improvement in HRQL following supplementation of anabolic steroids in patients with COPD. Our patients reported similar improvement in HRQL as measured by the Seattle Obstructive Lung Disease Questionnaire.

In our study, we used nutritional drink for nutritional intervention in the form of a commercially available high energy and high carbohydrate supplement. One 200 gm package of nutritional supplement drink costs about 5.40 Dollars approximately. Therefore, in the current study the total cost for the nutritional support per patient for the entire study duration was found to be approximately 120.00 Dollars.

Therefore, we confirm that cost-effectiveness of this intervention was efficient and that nutritional support in this study could be applied in clinical practice in India where incidence of malnutrition in COPD is quite high and most of the patients come from low socioeconomic background.

Our study had few limitations. Firstly, the sample size was small. A small difference between groups would not be detected as significant with small samples. A study with larger sample size and of longer duration of time to look into the benefits of nutritional intervention in underweight COPD patients is warranted.

Secondly, the nutritional intake was not standardized. Varying intake of dietary antioxidants also might have affected the results. It was not feasible to keep all the patients on a standardized diet in our study as all the patients were recruited from outpatient clinics. Another limitation was the potential bias arising from non-inclusion of female patients in the study. While we would have liked to include both male and female patients in comparable ratio, the number of male patients with COPD reporting to the outpatient clinic of the hospital far exceeded the number of female patients. This is possibly related to the fact that smoking in female subjects is much less common in India (40) and therefore, smoking-related COPD is much less prevalent.

## CONCLUSION

From the above observations, sufficient dietary energy and protein intake is essential to restore and maintain body weight, improve the exercise capacity and HRQL. Also, because of conflicting results from previous studies a definitive study of nutritional supplement is much needed by recruiting large number of malnourished patients, and supplementation should be continued for longer period of at least 3 months to allow time for possible muscle protein synthesis. Therefore, an assessment of dietary intake of protein and energy should be done in all patients with COPD. The available evidence is, however, not (yet) substantial enough to warrant dietary recommendations for primary prevention or management of COPD.
